# Use and Outcomes Associated With a Clear Prime for Infants Undergoing Cardiac Surgery

**DOI:** 10.1016/j.atssr.2026.02.024

**Published:** 2026-03-07

**Authors:** Brian Bateson, Brittany Ange, Bhargav Doddala, Emma Hazenberg, Lyubomyr Bohuta, James St. Louis

**Affiliations:** 1 Division of Pediatric Heart Surgery, Department of Surgery, Augusta University, Augusta, Georgia; 2 Division of Cardiothoracic Surgery, Department of Surgery, University of Washington, Seattle, Washington

## Abstract

**Background:**

The benefits of restricting blood transfusion at the time of cardiac surgery have been shown previously. Given the paucity of information in the pediatric population, we compared outcomes and use of a clear vs a blood prime.

**Methods:**

The Society of Thoracic Surgeons Congenital Heart Surgery Database was used to evaluate patients (2.5-12 kg) undergoing an index operation between January 1, 2016, and June 30, 2022 (versions 3.3 and 3.41). Demographics, intraoperative variables, and outcomes were investigated. Categorical variables were analyzed with χ^2^ tests, and Mann-Whitney *U* tests were calculated for continuous variables. Statistical significance was set at *P* < .0571.

**Results:**

During the study period, there were 60,037 patients with a blood prime and 7875 patients with a clear prime. The clear prime cohort was larger (weight, 7.3 kg vs 5.8 kg; body surface area, 0.4 m^2^ vs 0.3 m^2^) and older (380.2 days vs 198.0 days; all *P* < .0001. The blood prime group had more chromosomal abnormalities, nonchromosomal anatomic abnormalities, and any preoperative factor (all *P* < .0001). The clear prime cohort had a lower hematocrit, shorter time on a ventilator, shorter length of stay, and decreased complications (all *P* < .0001).

**Conclusions:**

Clear prime use has increased and was associated with a shorter time ventilated, extubation in the operating room, and decreased length of stay. Further studies are needed to identify opportunities to enhance blood conservation.

**In Short**

▪ The use of restrictive transfusion practices (clear prime) for neonates and infants has increased.
▪ There is some benefit to restrictive transfusion seen in early morbidity.

The benefit of restrictive blood transfusion in cardiac surgery has been shown in adult cardiac literature.^1^^,^^2^ Larger pediatric patients can routinely undergo cardiac surgery without receiving blood products, but smaller patients (2-12 kg) remain the largest challenge to this strategy.^3^ This can be attributed to circuit size, the volume needed to prime, and smaller circulating volumes. Some centers have been able to use miniaturized circuits as well as retrograde autologous priming to achieve this aim in this difficult patient population.^4^, ^5^, ^6^ The success of this approach is seen in shorter ventilation time, shorter intensive care unit length of stay, shorter total length of stay, and decreased overall morbidity.^6^^,^^7^

Success in the literature remains isolated to single-center retrospective reviews.^4^^,^^6^ Given the paucity of large-volume data comparing patients (2.5-12 kg) who have undergone cardiac surgery and received a blood prime (BP) for initiation of cardiopulmonary bypass (CPB) vs those that had a more restrictive approach with a clear prime (CP) for initiation of CPB, we used The Society of Thoracic Surgeons Congenital Heart Surgery Database (STS CHSD) to evaluate the national trend in CP by age and weight, complexity (by index case), and associated outcomes.

## Patients and Methods

The data for this research were provided by the STS National Database Participant User File Research Program. Patients included in this analysis were those with a weight between 2.5 and 12.0 kg undergoing elective or urgent cardiac surgery with the use of CPB from January 1, 2022, to June 30, 2022 (version 3.3, 3.41 of the STS CHSD). Exclusion criteria included weight outside of the previously stated range, a non-CPB case, emergency surgery, The STS—European Association for Cardio-Thoracic Surgery Congenital Heart Surgery (STAT) mortality category 5 patients, and nonindex procedures. This research was determined to be exempt research with a waiver of informed consent from the Advarra Institutional Review Board (Mod01760092, version 1.1; approval date, July 17, 2023).

### Variables

Variables provided by STS for this analysis included patient demographics, fundamental diagnosis, patient characteristics, syndromes, chromosomal abnormalities, noncardiac anatomic abnormalities, age and weight at the time of operation, CPB and cross-clamp times, initial hours on the ventilator, whether the patient was extubated in the operating room, postoperative length of stay, and major complications tracked by the database (ie chylothorax, neurologic deficit present at the time of discharge, unplanned cardiac reoperation, or unplanned cardiac catheterization, etc). Variables regarding blood product information included CP or BP, first hematocrit on bypass, last hematocrit on bypass, hematocrit after bypass, transfusion of blood products during or after the operation (outside of blood products used for a BP), and whether ultrafiltration was performed. Owing to the significant level of missingness for the types of blood products given and volume given, these variables were ultimately excluded from final analysis. The term “Transfused blood products during hospitalization” used here refers to blood products given after the initiation of CPB and excludes the prime.

### Statistical Analysis

Categorical variables are reported as frequency and percentage of incidence and were compared using the Pearson χ^2^ test to assess differences in the groups (CP vs BP). Continuous variables were compared using the Mann-Whitney *U* test. Data submissions with multiple procedural codes were not used for comparison between bypass prime groups. All analyses were performed using SAS 9.4 software (SAS Institute, Inc). The significance level was set at .05.

## Results

During the study period, 67,912 patients were eligible for review. Of these, 60,037 had a BP and 7875 had a CP. Table 1 summarizes the age and weight characteristics of the entire cohort.

**Table 1:** Age and Weight Characteristics

Characteristics	Clear Prime (n = 7875) No. (%)	Blood Prime (n = 60,037) No. (%)
Age, y		
0-1	5111 (60.9)	51,821 (86.3)
1-3	2397 (30.4)	7556 (12.6)
4-11	344 (4.4)	648 (1.1)
>11	22 (0.3)	10 (0.02)
Weight, kg		
2.5-4	1225 (15.6)	16,367 (27.3)
4-6	1669 (21.2)	19,365 (32.3)
6-8	1899 (24.1)	14,545 (24.2)
8-12	3082 (39.1)	9760 (16.3)

### Demographic Characteristics

Table 2 demonstrates the demographic characteristics of the cohort. The mean weight of the CP cohort was greater than the BP group, 7.3 vs 5.8 kg, respectively (*P* < .0001). The CP group had more patients with a syndrome, 33.5% vs 33.4% (*P* < .0001), but fewer with a nonchromosomal abnormality, 24.2% vs 24.5% (*P* < .0001), any chromosomal abnormality, 28.7% vs 30.2% (*P* < .0001), or any preoperative factors, 42.5% vs 46.1% (*P* < .0001) compared with the BP group.

**Table 2:** Preoperative Characteristics

Characteristics	Clear Prime (n = 7875)	Blood Prime (n = 60,037)	*P* Value
Age at surgery, mean (SD), d	380.2 (665.4)	198.0 (250.0)	
Sex			.8436
Male	4180 (53.5)	32,293 (53.8)	
Female	3637 (46.5)	27,737 (46.2)	
Weight, mean (SD), kg	7.3 (2.7)	5.8 (2.3)	
Body surface area, mean (SD), m^2^	0.4 (0.1)	0.3 (0.1)	
Prematurity			<.0001
Yes	1495 (19.0)	11,533 (19.2)	
No	6142 (78.0)	48,002 (80.0)	
Any syndrome			<.0001
No	5212 (66.2)	39,951 (66.5)	
Yes	2634 (33.5)	20,078 (33.4)	
Noncardiac anatomic abnormalities			<.0001
No	5940 (75.4)	45,307 (75.5)	
Yes	1906 (24.2)	14,722 (24.5)	
Any chromosomal abnormality			<.0001
No	5580 (70.9)	41,878 (69.8)	
Yes	2263 (28.7)	18,121 (30.2)	
Any preoperative factors			<.0001
No	4508 (57.2)	32,310 (53.8)	
Yes	3350 (42.5)	27,688 (46.1)	
STAT Mortality Category			<.0001
1	2232 (53.3)	17,893 (48.8)	
2	1784 (42.6)	16,753 (45.7)	
3	118 (2.8)	1236 (3.4)	
4	57 (1.4)	754 (2.1)	

Data are presented as n (%) unless indicated otherwise.

STAT, The Society of Thoracic Surgeons-European Association for Cardio-Thoracic Surgery Congenital Heart Surgery Mortality Categories.

### Operative Characteristics

Table 3 describes the operative characteristics of the entire cohort. The hematocrit was lower throughout the case in the CP group vs BP group measured as the first hematocrit on bypass (27.3% vs 31.6%), last hematocrit on bypass (31.5% vs 35.5%), and first hematocrit off bypass, (34.7% vs 37.1%; all *P* < .0001). The CP group received blood products less frequently during the hospitalization compared with the BP group at 78.9% vs 96.6%, respectively (*P* < .0001). The initial time on the ventilator was shorter in the CP group vs the BP group (36.2 hours vs 58.2 hours, respectively), with more in the CP group extubated in the operating room (42.9% vs 16.7%; both *P* < .0001).

**Table 3:** Operative and Postoperative Characteristics

Characteristics	Clear Prime (n = 7875)	Blood Prime (n = 60,037)	Missing Data	*P* Value
Cardiopulmonary bypass time, mean (SD), min	113.2 (67.8)	122.0 (71.0)		<.0001
Cross-clamp time, mean (SD), min	58.9 (48.0)	67.3 (49.4)		<.0001
Hematocrit				
First on bypass, mean (SD), %	27.3 (6.4)	31.6 (5.2)	4450	<.0001
Last on bypass, mean (SD), %	31.5 (6.6)	35.5 (6.2)	4625	<.0001
After bypass, mean (SD), %	34.7 (6.8)	37.1 (6.1)	7398	<.0001
Transfused blood products during hospitalization			7806	<.0001
Yes				
No	5629 (78.9)	51,171 (96.6)		
Ultrafiltration			40,060	<.0001
No	355 (11.7)	5788 (23.3)		
Yes	2687 (88.3)	19,022 (76.7)		
Initial time on ventilator, mean (SD), h	36.2 (114.4)	58.2 (141.4)	6248	<.0001
Extubate in operating room			2429	<.0001
Yes	3303 (42.9)	9652 (16.7)		
No	4403 (57.1)	48,125 (83.3)		
Postoperative length of stay, mean (SD), d	14.4 (26.3)	18.3 (32.1)		<.0001
Complication				<.0001
No	4138 (52.6)	22,916 (38.2)		
Yes	3661 (46.5)	36,594 (61.0)		

Data are presented as n (%) unless indicated otherwise.

### Prime Use by Specific Procedure

Table 4 lists bypass prime strategy by specific procedure. Ventricular septal defect and tetralogy of Fallot repair were the procedures where a CP was used most frequently (29.7% and 17.8%, respectively).

**Table 4:** Prime Use by Major Index Procedure

Procedure	Clear Prime (n = 4191) No. (%)	Blood Prime (n = 36,636) No. (%)
VSD repair	1247 (29.7)	10,384 (28.3)
Tetrology of Fallot repair	747 (17.8)	7117 (19.4)
Pulmonary atresia/VSD/MAPCA	51 (1.2)	442 (1.2)
CAVSD repair	504 (12.0)	4737 (12.9)
Glenn	577 (13.8)	3123 (8.5)
Fontan	238 (5.7)	392 (1.01)
Ebstein’s repair	31 (0.7)	190 (0.5)
Arterial switch operation	311 (7.4)	4174 (11.4)
Truncus arteriosus repair	57 (1.4)	754 (2.01)
Double-outlet right ventricle repair	118 (2.8)	1236 (3.4)
Aortic arch repair + VSD repair	310 (7.4)	4087 (11.2)

CAVSD, complete atrioventricular septal defect; MAPCA, multiple aortopulmonary collateral arteries; VSD, ventricular septal defect.

## Comment

This analysis provides an important update on national benchmarks for transfusion rates among patients undergoing heart surgery from the prior study in 2018.^3^ In the previous STS CHSD study, looking at intraoperative transfusion of packed red blood cells, the transfusion rate was nearly 100% across all case complexity for patients aged 0 days to 1 year.^3^ Results from this study for the same age range show that nearly 9% of patients receive a CP, with 20.8% of the entire CP cohort being discharged without having received any transfusion. Although not a seismic shift in blood conservation in pediatric cardiac surgery, it signals an important trend in the approach to blood conservation in a difficult patient population.

A prior study showed that greater intraoperative and postoperative transfusion rates were associated with a prolonged duration of mechanical ventilation.^8^ Surgical programs in Berlin and Seattle have published their approach to circuit management as well as intraoperative thresholds for transfusion to maintain adequate hematocrit and perfusion while on bypass.^5^^,^^7^ These same groups have demonstrated that transfusions increased the time of mechanical ventilation and intensive care unit length of stay.^6^^,^^7^

The findings in this study confirm prior research. The CP group was more likely to be extubated in the operating room, have less time of mechanical ventilation, and decreased total length of stay postoperatively. Even though most patients in the CP group do receive some form of transfusion in the operating room (∼79%), the BP group receives a transfusion 96.6% of the time in addition to the BP. There is some benefit to restrictive transfusion seen in early morbidity. Ultimately, how to implement a national approach to restrictive blood transfusion remains elusive due to the heterogeneity of practice by individual surgical programs and surgeons as well as the diverse spectrum of congenital heart disease.

### Limitations

This study has several limitations that warrant mention. There were a significant number of case submissions with incomplete information regarding transfusion of blood products at the different time points tracked by the STS CHSD, which ultimately affects the power of the present study. This also meant an analysis of the different time points (first 24 hours, after 24 hours) of transfusion could not be completed.

We cannot account for center variability regarding CPB circuit setup, which may allow for some programs to do a CP whereas others simply cannot due to hemodilution from a larger circuit.

We also did not control for the use of antifibrinolytics or procoagulant medications that can vary widely from center to center and can account for some success in leaving the operating room without a transfusion.

Lastly, this analysis cannot account for transfusion guidelines that are unique to individual centers and can influence the results seen in these data.

### Conclusions

There has been an increase in the use of restrictive transfusion practices with encouraging results for neonates and infants who receive a CP as part of their surgery. Further study is warranted to identify ways to standardize this practice of blood conservation at a programmatic and national level to continue to reduce unnecessary exposure to blood products and improve outcomes.
